# *In vitro *cell cultures obtained from different explants of *Corylus avellana *produce Taxol and taxanes

**DOI:** 10.1186/1472-6750-6-45

**Published:** 2006-12-06

**Authors:** Federica Bestoso, Laura Ottaggio, Andrea Armirotti, Alessandro Balbi, Gianluca Damonte, Paolo Degan, Mauro Mazzei, Francesca Cavalli, Bernardetta Ledda, Mariangela Miele

**Affiliations:** 1 Laboratory of Plant and Pharmaceutical Biotechnology, Advanced Biotechnology Center (ABC), Largo R. Benzi, 10, 16132 Genova, Italy; 2 Department of Translational Oncology, National Cancer Research Institute, IST, Largo R. Benzi, 10, 16132 Genova, Italy; 3 DIMES: Department of Experimental Medicine and Center of Excellence for Biomedical Research, University of Genova, Viale Benedetto XV, 7, 16132 Genova, Italy; 4 Department of Pharmaceutical Sciences, University of Genova, Viale Benedetto XV, 3, 16132 Genova, Italy

## Abstract

**Background:**

Taxol is an effective antineoplastic agent, originally extracted from the bark of *Taxus brevifolia *with a low yield. Many attempts have been made to produce Taxol by chemical synthesis, semi-synthesis and plant tissue cultures. However, to date, the availability of this compound is not sufficient to satisfy the commercial requirements. The aim of the present work was to produce suspension cell cultures from plants not belonging to *Taxus genus *and to verify whether they produced Taxol and taxanes. For this purpose different explants of hazel (*Corylus avellana *species) were used to optimize the protocol for inducing *in vitro *callus, an undifferentiated tissue from which suspension cell cultures were established.

**Results:**

Calli were successfully induced from stems, leaves and seeds grown in various hormone concentrations and combinations. The most suitable callus to establish suspension cell cultures was obtained from seeds. Media recovered from suspension cell cultures contained taxanes, and showed antiproliferative activity on human tumour cells. Taxol, 10-deacetyltaxol and 10-deacetylbaccatin III were the main taxanes identified. The level of Taxol recovered from the media of hazel cultures was similar to that found in yew cultures. Moreover, the production of taxanes in hazel cell cultures increased when elicitors were used.

**Conclusion:**

Here we show that hazel cell cultures produce Taxol and taxanes under controlled conditions. This result suggests that hazel possesses the enzymes for Taxol production, which until now was considered to be a pathway particular to *Taxus *genus. The main benefit of producing taxanes through hazel cell cultures is that hazel is widely available, grows at a much faster rate *in vivo*, and is easier to cultivate *in vitro *than yew. In addition, the production of callus directly from hazel seeds shortens the culture time and minimizes the probability of contamination. Therefore, hazel could become a commercial source of Taxol and taxanes, both to be used as new therapeutic agents or as new precursors for Taxol semi-synthesis.

## Background

Taxol (generic name paclitaxel), a potent antimitotic drug employed for the treatment of a variety of cancers [1,2], was originally extracted from the bark of *Taxus brevifolia*, a slow growing yew native to the North-Western Pacific area [3]. Taxol was found to have a unique way of preventing the growth of cancer cells, through inhibiting microtubule dissociation, due to its binding affinity to tubulin [4].

The major limitation encountered in the extensive use of this drug is its short supply, since *T. brevifolia*, as well as other *Taxus *spp, yields very low amounts of Taxol. Moreover, extraction of this compound from the intact plant generally requires labour intensive procedures, besides posing environmental concerns. In the last few years the increasing application in additional cancer types, and its early use in the course of the therapy, has prompted intense efforts to meet the demand for Taxol. The total synthesis of this compound on an industrial scale seemed economically unrealistic, because of the complex structure of the molecule [5-7]. Presently, most of the drug for clinical use is produced by semi-synthesis [8], starting from a natural precursor, 10-deacetylbaccatin III, which can be obtained from differentiated yew tissues, mainly leaves, with a relatively good yield [9,10]. Semi-synthesis is also used to produce Taxotere, a synthetic analog with antitumour activity similar to Taxol [8]. However, the semi-synthetic approach suffers from some limitations, relied upon the extraction and isolation of the precursor from differentiated tissues, which can vary significantly in yield, depending on epigenetic and environmental factors [11,12].

Plant cell cultures are, therefore, considered one of the most promising approaches to provide a stable supply of Taxol and related compounds, generally named taxanes. Several attempts have been made to produce cell cultures from different *Taxus *spp [13-15], and various elicitors have been used to increase the productivity and the release of Taxol into culture medium [16-20]. However, the availability of this drug is still limited and its cost is very high, mainly due to the recalcitrant behaviour of *Taxus *spp under *in vitro *conditions. Efforts have also been focused on the isolation and cultivation of Taxol-producing endophytic fungi associated with yew, but the Taxol yield was relatively small compared to the tree [21-23].

Thus, *Taxus *spp and endophytic fungi have been considered the only sources to be exploited for the commercial supply of Taxol and taxanes. Hazel has also been reported as a Taxol-producing species through bioprospection among angiosperms [24,25]. However, it is not realistic to consider the possibility of extracting Taxol from these plants, both for the low amount recovered (1/10 the level of yew), and for the need of extracting the compound from the intact plant. Moreover, Taxol found in hazel was actually thought to derive from endophytic fungi living inside the hazel, rather than from hazel itself [24].

Knowledge of the biosynthetic pathway is another important step for improving the production of this drug. The regulation of Taxol biosynthesis by overexpressing selected genes in yew, or other suitable hosts, can potentially address the supply issue [26-28].

The objective of the present work was to define the experimental conditions for obtaining suspension cell cultures of hazel (*Corylus avellana *species), and to verify whether they produced Taxol and taxanes. Hazel cell culture systems could represent a potential source of Taxol and related compounds, for which the extraction from intact plant or the chemical synthesis is not commercially feasible. Calli from differentiated tissues of *C. avellana *and *T. baccata *were produced in order to establish cell cultures. Cell culture media were screened for the presence of taxanes and tested for their biological activity on a human cell line. Our results showed that taxane production in hazel and yew cell cultures was similar, however *in vitro *cultivation of hazel, in particular starting from seeds, were more advantageous than *in vitro *cultivation of yew. Moreover, the production of taxanes by hazel cells was inducible by elicitors.

## Results and discussion

### Callus derived from hazel seeds was precocious and fast-growing

**Figure 1:**
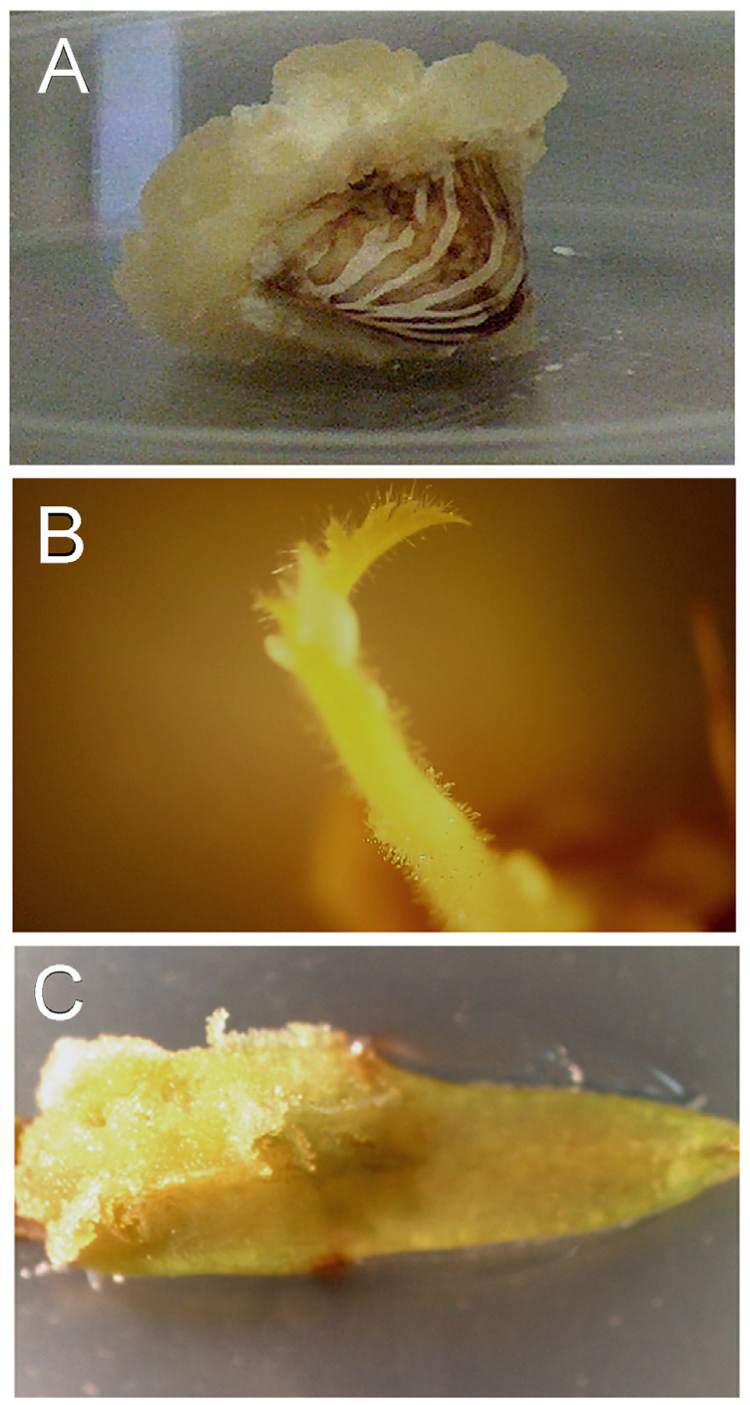
**Callus induction**. Callus from hazel seed (A), somatic embryo from hazel seed (B) callus from yew leaf (C).

To optimize the initiation protocol for *in vitro *cultivation of hazel, different explants were used to establish callus cultures. Calli were successfully induced from stems, leaves and seeds cultured on media supplemented with several concentrations and combinations of plant growth regulators. Callus formation was generally precocious, developing actively from the 10^th^–30^th ^day of culture, and showed a large variability with respect to the phytohormone concentration and to the type of explant. Table 1 shows the percentage of callus derived from different explants, after 30 days of culture. Almost all the phytohormone combinations were able to induce callus, with a preference for 2,4-dichlorophenoxyacetic acid (2,4-D), when used alone or in association with Benzyladenine (BA). The induction of seed derived callus was also high (more than 50%) when Naphthalene Acetic Acid (NAA) in association with BA was used. The best sources of explants were stems and seeds, with a maximum of callus induction of 75%. In addition, calli derived from seeds remained white and friable (Fig. 1A) over a period of about two years, with a regular growth rate (169 ± 15 mg/week). Calli derived from stems and leaves declined after 8-10 months following the initial subculture, resulting in a very slow-growing, brown coloured callus, dying 1-2 months later. Part of the seed explants, cultured in medium supplemented with plant growth regulators for callus induction, produced somatic embryos after several days of culture (Fig. 1B), with a maximum of somatic embryo formation at 14-18 days of culture (Table 1). Somatic embryo development was not apparently dependent on the phytohormone concentration/combination; however, seed explants grown in medium without phytohormones did not develop embryos. Somatic embryos, transferred into medium without phytohormones and maintained in a phytochamber, produced *in vitro *plantlets that are nowadays maintained in a greenhouse.

In parallel, the same type of explants from *T. baccata *were used to establish callus cultures, as positive control. Callus formation from yew explants exhibited generally slower growth occurring from the 30^th ^to 60^th ^day of culture. Table 2 shows yew callus induction after 60 days of culture. The most productive explants were stems and leaves (Fig. 1C), with a maximum callus induction of 76% and 63%, respectively, when grown in 2,4-D. Seed derived calli were less productive, reaching a maximum of 52% when grown in NAA. These calli remained yellow pale and friable for about one year with a very slow growth rate, while stem and leaf callus became brown and died after 10-12 months of culture. Callus from *T. baccata *stems and leaves have already been produced using different combination of growth regulators [29,30]. Some attempts have already been made to produce callus from yew seeds. However, seeds were first germinated *in vitro*, and embryos or seedlings were used for establishing callus cultures [31,32]. Here, we directly transferred sterilised hazel and yew seeds into a medium suitable for callus induction, without waiting for seedling germination. This new approach offered some advantages, including the shortening of the time necessary for callus appearance. In addition, cultures initiated from seeds also minimized the probability of contamination as compared to the culture obtained from stems and leaves.

### Hazel suspension cell cultures produced taxanes

Cell suspension cultures of *C. avellana *were established from selected white and friable seed derived calli, 2 months old, grown in medium supplemented with 3 different hormone concentrations and/or combinations. In comparison, cell cultures from stem derived callus of *T. baccata *were prepared and grown in 2 mg/l NAA, a growth condition already shown to be suitable for the production of yew cell cultures [33,34]. Despite a certain variability, hazel and yew cultures have been found to produce taxanes in similar level, and to release them in the medium over a period of 16 weeks, as shown by ELISA analysis. Although ELISA did not permit either to identify or to quantify the single taxane, it is generally used to screen for the presence of taxanes [35,24]. No correlation with hormone concentration and/or combination and taxane production was observed. However, the present study was mainly aimed at verifying whether hazel, in controlled conditions, such as *in vitro *culture, was able to produce taxanes, and whether this production was comparable with yew cell culture. Further studies are needed in order to investigate the main environmental factors that influence the production of taxanes in hazel cells. In addition, the type of explants from which the callus was obtained could also be of importance for the production of these metabolites. In the present study only seed derived calli were used to establish cell cultures, due to their higher productivity and faster growth.

### Taxane production in hazel cell cultures was not due to fungus contamination

To verify whether taxanes found in hazel cultures could be attributed to the presence of endophytic fungi, samples of callus and cell culture medium, maintained in sterile conditions for at least 6 months, were grown in a substrate specific for fungal growth. No hyphal growth was observed in plates containing either callus or medium, examined periodically by microscope, for a period of 2 months. This result indicated that taxanes recovered from hazel culture was to be ascribed to hazel metabolism rather than to be a consequence of endophytic fungus contamination, as suggested [24].

### Hazel cell culture extracts induced mitotic block

**Figure 2:**
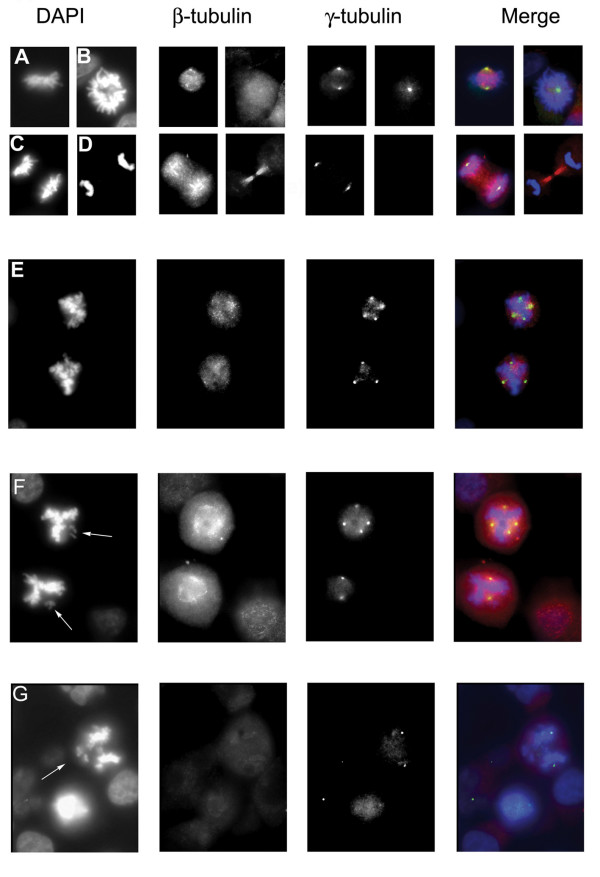
**Effects of hazel and yew cell culture extracts on mitosis**. DNA was visualised with DAPI (blue), mitotic spindles with a β-tubulin antibody (red) and centrosomal structures with a γ-tubulin antibody (green-yellow). Mock treated cells (*A-D*); 10 nM Taxol treated cells (*E*); hazel medium extract treated cells (*F*); and yew medium extract treated cells (*G*). Normal metaphases (*A, B*); anaphases (*C*); and telophases (*D*) were mainly found in mock treated cells. Multipolar metaphases (*E, F*) and multipolar metaphases or anaphases with displaced chromosomes (*F, G *arrows) were mainly found in treated cells.

Extracts from hazel and yew culture media were evaluated for their effects on cell proliferation of human tumour SK-Mes-1 cells. Table 3 shows the effects of media collected from hazel and yew cultures recovered after 30 days of culture. A 10 nM Taxol treatment was also included as a positive control. Previous experiments (data not shown) treating SK-Mes-1 cells with different amounts of Taxol, ranging from 0.2 nM to 10 μM, indicated that 10 nM was the most suitable dose to identify a change in the anaphase/metaphase ratio. Higher doses caused the complete block in metaphase with a lack of anaphases, preventing a precise evaluation of the ratio. An increase in mitotic index and a decrease in the anaphase/metaphase ratio were observed in Taxol and hazel extract treated cells. The increase in mitotic index was mainly due to the block of the metaphase to anaphase transition, with the consequent inhibition of cell proliferation. Cells incubated with hazel extract showed the highest mitotic index and the lowest anaphase/metaphase ratio. This indicated that hazel extract exhibits a stronger biological effect than 10 nM Taxol. Yew extract showed a slight effect on the anaphase/metaphase ratio, without affecting the mitotic index. Multipolar metaphases and metaphases with displaced chromosomes, were also observed in all treated cells (Fig. 2). Together, these results suggested that taxanes contained in hazel and yew extracts were able to act on mitotic spindle, probably by binding to tubulin, and that the amount of Taxol-like compounds contained in hazel extract was higher than in yew extract.

### HPLC-MS identified Taxol and other taxanes in hazel media cell cultures

**Figure 3:**
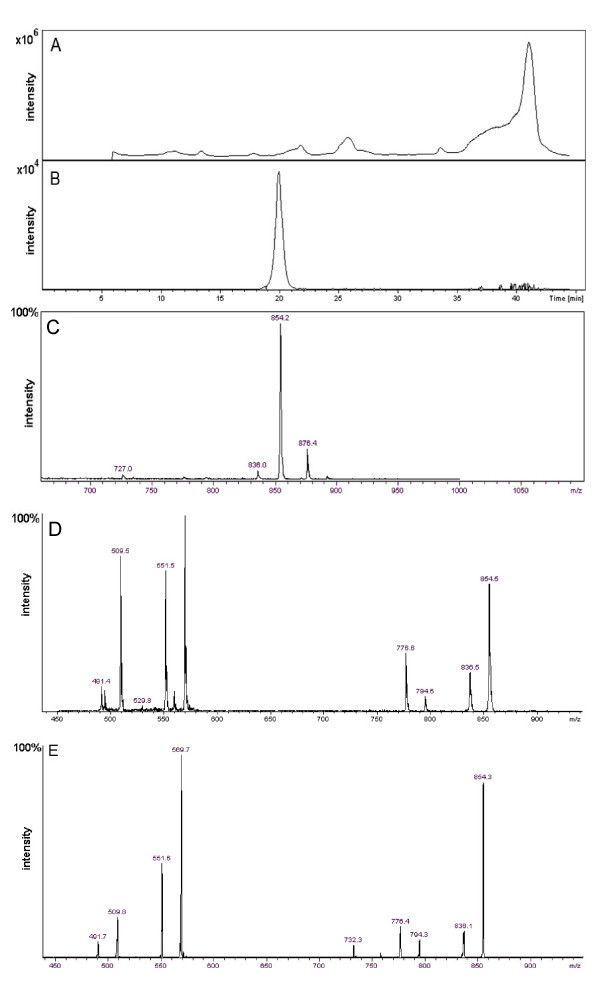
**HPLC-MS analysis of medium recovered from hazel cell culture**. Total ion chromatogram (*A*), extracted ion current (*B*) and mass spectrum of taxol (*C*); MS/MS spectra of the sample (*D*) and standard taxol (*E*).

In order to identify the main taxanes contained in hazel cultures, media recovered from 30 day old cultures, already shown to be positive for the presence of taxanes by ELISA, were analysed by RP-HPLC-MS. Total ion chromatogram (Fig. 3A), extracted ion chromatogram (Fig. 3B), MS (Fig. 3C) and MS/MS (Fig. 3D) spectra of Taxol recovered from the supernatant of one of the hazel cell cultures, are shown. MS/MS spectrum of standard Taxol is shown in Fig. 3E. Hazel cell culture media were found to contain mainly Taxol (up to 1 mg/l), 10-deacetyltaxol (up to 1.8 mg/l), and 10-deacetylbaccatin III (up to 0.5 mg/l). Baccatin III and 7-xylosyltaxol were also identified, but not precisely quantified, due to the low level of these taxanes, under the HPLC-MS quantification limit. In particular, hazel medium extract used to test its biological effect on SK-Mes-1 cells contained 17 μg/ml of Taxol. We could then estimate that SK-Mes-1 cells received the equivalent of 82 nM Taxol. This value was in keeping with the ability to block the metaphase/anaphase transition, higher in hazel extract versus both Taxol and yew extract treatments (Table 3). It is worth noting that the yew extract used for evaluating the biological activity, despite its positivity with ELISA, was not found to contain Taxol when analysed with HPLC-MS, probably due to the low level of Taxol in this sample. Although there was a certain variability, these results confirmed that hazel cell cultures were able to produce Taxol with a yield comparable with what was obtained from yew cell cultures without elicitation [18,36,37]. Studies are in progress to investigate the main factors that influence the production of taxanes in hazel cells, with the aim of selecting stable hazel cell lines with high productivity of Taxol.

### Taxanes produced in hazel cell cultures was increased through elicitation

**Figure 4:**
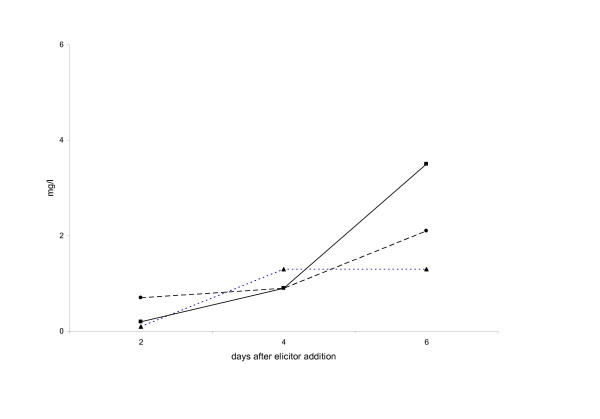
**Taxane production after elicitation**. Taxane recovered in the medium of hazel suspension cultures after 2, 4 and 6 days of elicitation.  control;  treatement with 200 μM methyljasmonate;  treatment with 200 μM methyljasmonate and 182 μg/ml chitosan. Taxanes considered are: Taxol, 10-deacetylbaccatin III, Baccatin III, 10-deacetyltaxol, 7-xylosyltaxol.

Hazel suspension cultures from seed derived calli were elicitated with methyljasmonate or methyljasmonate plus chitosan. The medium was recovered from these cell cultures 2, 4 and 6 days after elicitation, and analysed by HPLC. The production of total taxanes, including Taxol, 10-deacetylbaccatin III, baccatin III, 10-deacetyltaxol, 7-xylosyltaxol, increased after 6 days of elicitation (Fig. 4). Treatments with methyljasmonate plus chitosan were more effective in increasing taxane production than those using methyljasmonate alone. Similar effects have been reported for yew cells [38]. These data suggest that a longer period of elicitation could be a good way to obtain greater production of Taxol and related compounds from hazel cell cultures.

## Conclusion

The finding of Taxol in certain hazel varieties was emphatically reported [25] and gave hope for the employment of new species for Taxol production. However, it was thought that at least some, and perhaps all, of the Taxol present in hazel derived from endophytic fungi living inside hazel, rather than produced by hazel metabolism itself [24]. Here we show, for the first time, that hazel can produce Taxol when it is in aseptic conditions, likely in the absence of microorganisms such as endophytic fungi. We can then say that, in all probability, hazel possesses the metabolic pathway for Taxol production. Therefore, further biochemical studies might be addressed to identify in hazel, the enzymes involved in Taxol biosynthesis, till now considered to be a particular metabolism of *Taxus *spp among higher plants. Characterization of these enzymes is also an essential step for bioengineering hazel cell cultures. Engineering of either Taxol-producing or non Taxol-producing organisms is considered a promising approach for the future availability of this drug. Many *Taxus *spp have been explored for producing Taxol through cell cultures; however, the demand of this compound has not yet been satisfied. The use of suspension cell cultures, obtained from a faster-growing and widely diffuse plant such as hazel, is more promising than yew cell culture used so far. The present work shows that hazel cell cultures are able to yield taxanes in a similar level as yew cell cultures, and that *in vitro *cultivation of hazel is more advantageous with respect to *in vitro *cultivation of yew. In particular, the production of callus directly from hazel seeds reduces culture time, partially overcoming the somaclonal variation. Another advantage of using this new approach is that *in vitro *cultures initiated from seeds minimize the probability of contamination as compared to those obtained from other differentiated tissues, such as leaves and stems. We can then conclude that, by developing appropriate methodology, hazel species could become a new commercial source of Taxol and taxanes, both to be used as new therapeutic agents or as new precursors for Taxol and/or Taxotere semi-synthesis.

## Methods

### Plant material and callus induction

Stems, leaves and seeds derived from adult *C. avellana *trees cultivated in 4 Italian towns (Carcare, Ventimiglia, Genova, Rapallo), differing in climate and height, were used for callus induction. Young branches, collected from April to July were rinsed with tap water, sterilized with 5% NaClO for 20 min and rinsed 3 times with sterile distilled water. Stems were separated from leaves and transferred to plates containing 10 g/l agar Murashige and Skoog medium [39], pH 5.5, supplemented with 1× vitamins (Sigma), 20 g/l sucrose and phytohormones: 2,4-D, BA and NAA at different concentrations and combinations (Table 1). From the same trees, immature seeds were collected from August to December, depending on climate and height. Shells were removed and seeds were sterilized with 5% NaClO for 20 min, rinsed 3 times with sterile distilled water, cut into 4 fragments with a sterile blade and cultured in the same conditions as leaves and stems. Part of the sterile seed pieces were also transferred into medium without phytohormones, for inducing embryo germination. One experiment consisted of at least 24 plates, containing 8 different concentrations and/or combinations of phytohormones for each tissue types (stems, leaves and seeds). Each plate contained about 8 fragments from the different tissues. Seed fragments were also transferred into 5 plates without phytohormones. Fragments were maintained under darkness in a chamber growth at 26 ± 1°C. Callus induction and embryo formation were evaluated after 15 days. The mean was calculated on at least 4 experiments for each tissue type. Experiments were performed using 4 different hazel plants. Calli were periodically weighed and transferred into fresh medium, supplemented with phytohormones and subcultured every 15 days. Stems and leaves from *T. baccata *were also prepared and cultured as described for hazel explants. Sterile mature seeds bereft of aril were longitudinally excised and transferred into medium with phytohormones. Callus formation was evaluated after 30 days of culturing. Callus induction means were calculated on 3 experiments for each tissue type. Hazel embryos were transferred into magenta flasks with medium without phytohormones and maintained in a chamber growth at 26 ± 1°C with 16 light and 8 dark photoperiod. Two months later young plantlets were transferred into soil and maintained in a greenhouse.

### Cell cultures

The most productive seed derived calli, 2 months old, were used for establishing cell suspension cultures of *C. avellana *species. About 2 g of white and friable callus, grown in solid medium supplemented with 2,4-D 0.5 mg/l, 2,4-D 1 mg/l and 2,4-D 1 mg/l plus BA 0.5 mg/l, were transferred to 50 ml of the same medium lacking agar. At least 4 flasks for each hormone combination were prepared for 4 different experiments. The suspension cultures were maintained in Erlenmeyer flasks at 26 ± 1°C in the dark, on orbital shaker at an agitation speed of 125 rpm, and subcultured every 2 weeks. Before transferring cells into fresh medium, 4 ml of suspension culture were centrifuged and supernatant processed for taxane analysis, for a period of 16 weeks. In parallel, suspension cell cultures from *T. baccata *callus were performed and used as positive control. Particularly, about 2 g of stem derived callus, grown in solid medium supplemented with NAA 2 mg/l were transferred into 50 ml of the same medium lacking agar, and used for establishing cell cultures. Three Erlenmeyer flasks were prepared and maintained at the same growth condition as hazel cells. Before transferring cells in fresh medium 4 ml of suspension culture were centrifuged and supernatant processed for taxane analysis, as above.

### Extract preparation

Extracellular taxanes were extracted from 4 ml of supernatant from hazel and yew suspension cultures by filtering through a 0.2 μm Gelman nylon membrane filter [40]. Then, 300 μl of methanol was passed through the filter to elute the adsorbed Taxol from the nylon membrane and collected into a 2.0 ml glass vial.

### ELISA

A competitive solid phase immunoassay TAO1 (Hawaii Biotech Inc) was employed for the detection of taxanes. Free taxanes present in methanol hazel and yew culture medium extracts were detected by inhibition of the reaction between solid phase Taxol-protein conjugate and anti-taxane antibody. Assay was performed following the procedures recommended by suppliers, using a 96 well flat bottom plates (Corning Inc, Costar) coated with Taxol-protein antigen. The plates were blocked with 1% (w/v) bovine serum albumin (Sigma) in phosphate buffer solution and excess antigen was washed away. Solid phase bound Taxol was incubated with 50 μl of medium extracts, Taxol standard and a specific anti-taxane rabbit antibody. Antibody bond to the solid-phase-bond Taxol was detected by using an alkaline-phosphatase-conjugated secondary antibody, using alkaline phosphatate substrate, p-nitrophenyl phosphate (Sigma), as chromophore-generating substance. Absorbance of each well was read on a dual wavelength Mithras LB 940 ELISA reader (Berthold Technologies). Taxanes were determined in each sample using a curve plot generated with Taxol standards. Samples with values higher then 0.5 ng/ml were considered positive for the presence of taxanes.

### Fungus growth evaluation

About 2 g of callus fragments or 1 ml of suspension cell culture derived from the same kind of callus, were transferred to plates containing Saboraud Dextrose Agar (Fluka). Six agar plates with 4 callus fragments each and 6 plates with suspension cultures were incubated at 26 ± 1°C for 8 weeks. Plates were examined weekly using a Stemi SV6 (Zeiss) stereomicroscope to evaluate the possible fungal growth.

### Indirect Immunofluorescence

SK-Mes-1 cells (a squamous cell lung carcinoma cell line, purchased from American Type Culture Collection) were used to evaluate the effects of hazel and yew medium extracts on mitotic microtubules. Fifty μl of extract to be tested were added to cells plated on Lab-Tek chamber slides (Nalge Nunc) and incubated overnight. The microtubule, centrosomal and nuclear structures were detected by indirect immunofluorescence as described [41]. Centrosomes were visualized by using a rabbit polyclonal antibody to γ-tubulin (Sigma) and mitotic microtubules with a mouse monoclonal antibody to β-tubulin (BD Pharmingen) in the same immunocytochemical reaction. The slides were washed, stained with 0.1 μg/ml 4',6-Diamidino-2-phenylindole (DAPI), examined and images aquired using a Provis (Olympus) fluorescence microscope with a digital CCD (Charge Coupled Device) camera. Mitotic indices represent counts of at least 1000 interphase cells from 3 independent experiments; anaphase/metaphase ratio was evaluated counting at least 50 mitotic cells from 3 independent experiments.

### HPLC-MS and HPLC analysis

For taxanes identification, samples were analysed by an Agilent 1100 HPLC system coupled to a 1100 MSD Ion Trap mass spectrometer, equipped with an electrospray ion source. The column was an Agilent Zorbax C18 (0.5 × 100 mm) and the gradient was a standard water:acetonitrile (ACN) from 30% to 100% in 40 min. Both water and ACN were added with 0.1% formic acid. Mass spectra were acquired in positive ion mode. The analysed data were qualified against the following standards (Hauser): Taxol, 10-deacetylbaccatin III, baccatin III, 10-deacetyltaxol, 7-xylosyltaxol, which were prepared in methanol. Taxol and taxanes were quantified by Waters Associated Model 600 HPLC system equipped with Waters Associated Model 500 UV detector at 227 nm using a Phenomenex Curosil G (250 × 4.6 mm). The mobile phase was water:ACN (55:45) at 1.0 ml/min. The analysed data were quantified against the above standards.

### Elicitation

Six different suspension cell cultures were prepared with hazel callus induced in medium supplemented with 2,4-D 0.5 mg/l plus BA 1 mg/l. About 2 g of seed derived callus were transferred into Erlenmeyer flasks containing 60 ml of liquid medium with the same phytohormone concentrations and combinations as callus induction. After 1 week, each culture was divided into 3 different flasks at which 30 ml of fresh medium were added. Eight days later, elicitors were added. Six flasks were treated with 200 μM methyljasmonate (Sigma) ethanol solution; 6 flasks were treated with 200 μM methyljasmonate ethanol solution together with 0.182 μg/l chitosan (AgriHouse Inc) aqueous solution, according to Linden [38]. The remaining 6 flasks were mock treated with ethanol and used as control. Two, 4 and 6 days after elicitation 5 ml from each suspension culture were collected, centrifuged, extracted as above and analysed for the presence of taxanes by HPLC.

## Authors' contributions

FB carried out the experimental work related to the establishment of *in vitro *cultures and participated in the design of the study. LO carried out immunoflorescence analysis and participated in drafting the manuscript. AA an GD carried out HPLC-MS analysis. AB and M Mazzei participated in the design of the study. PD carried out immunassays. FC carried out elicitation experiments. BL participated in the establishment of *in vitro *cultures. M Miele conceived the study, drafted the final manuscript and participated in all stages of the work. All authors read and approved the final manuscript.

## Figures and Tables

**Table 1:** Callus induction (A) and somatic embryo production (B) from *C. avellana *explants

Hormone concentration and combination (mg/l)	A*			B**
	
	Leaves	Stems	Seeds	Seeds
2,4-D (1)	35.5 ± 33.4	68.5 ± 40.9	56.2 ± 37.3	15.5 ± 11.9
2,4-D (0.5)	41.9 ± 26.4	75.0 ± 50.0	61.6 ± 23.3	15.8 ± 12.7
BA (1)	4.6 ± 6.3	15.0 ± 30.0	13.8 ± 22.3	8.7 ± 18.0
BA (0.5)	4.6 ± 8.6	0	10.3 ± 19.3	13.4 ± 11.7
2,4-D (0.5) + BA (1)	45.2 ± 27.9	58.3 ± 50.1	75.0 ± 31.6	15.0 ± 12.5
2,4-D (1) + BA (0.5)	43.7 ± 29.8	75.0 ± 50.0	72.2 ± 26.6	17.1 ± 16.5
NAA (2)	22.1 ± 20.3	5.4 ± 10.8	41.3 ± 28.0	14.4 ± 17.3
NAA (2) + BA (0.5)	38.2 ± 29.8	33.3 ± 34.0	63.2 ± 39.6	16.2 ± 13.9

*number of explants producing callus/total number of explants after 30 days of culture time.

**number of explants producing somatic embryos/total number of explants after 15 days of culture time.

All the values represent the mean ± SD from 4 different experiments using 4 different plants.

**Table 2:** Callus induction from *T. baccata *explants

Hormone concentration and combination (mg/l)	A *		
	
	Leaves	Stems	Seeds
2,4-D (1)	56.3 ± 36.8	76.3 ± 33.0	37.7 ± 16.0
2,4-D (0.5)	63.8 ± 37.8	65.8 ± 28.7	43.7 ± 8.1
BA (1)	3.0 ± 6.0	2.8 ± 4.1	0
BA (0.5)	4.5 ± 10.1	9.0 ± 9.5	32.7 ± 6.7
2,4-D (0.5) + BA (1)	8.0 ± 10.5	24.3 ± 10.3	21.0 ± 16.4
2,4-D (1) + BA (0.5)	30.3 ± 18.2	32.5 ± 25.3	16.0 ± 9.3
NAA (2)	33.0 ± 22.4	56.3 ± 39.9	52.0 ± 24.8
NAA (2) + BA (0.5)	10.0 ± 2.0	12.0 ± 15.7	13.0 ± 5.2

*number of explants producing callus/total number of explants after 60 days of culture time.

All the values represent the mean ± SD from 3 different experiments using 2 different plants.

**Table 3:** Effects of hazel and yew medium extracts on mitotic index and metaphase/anaphase transition

	Mitotic index	Anaphases/metaphases no./no.	p*
Control	0.015 ± 0.01	0.189 ± 0.10	-
Taxol (10 nM)	0.040 ± 0.02	0.028 ± 0.02	<0.001
Hazel extract	0.065 ± 0.02	0.007 ± 0.01	<0.001
Yew extract	0.017 ± 0.01	0.071 ± 0.01	<0.01

Data are the mean ± SD of at least 3 experiments. For each experiment at least 1000 interphase nuclei and 50 mitosis were counted.

*p value estimates of significance by Fisher's exact test on anaphase/metaphase ratio.
